# Can Certain Genotypes Predispose to Poor Asthma Control in Children? A Pharmacogenetic Study of 9 Candidate Genes in Children with Difficult Asthma

**DOI:** 10.1371/journal.pone.0060592

**Published:** 2013-04-03

**Authors:** Basima Almomani, Ahmed F. Hawwa, Jeffrey S. Millership, Liam Heaney, Isabella Douglas, James C. McElnay, Michael D. Shields

**Affiliations:** 1 Clinical and Practice Research Group, School of Pharmacy, Queen's University Belfast, Belfast, United Kingdom; 2 Centre for Infection and Immunity, School of Medicine, Dentistry and Biomedical Sciences, Queen's University Belfast, Belfast, United Kingdom; 3 The Royal Belfast Hospital for Sick Children, Belfast Health and Social Care Trust, Belfast, United Kingdom; Yale School of Public Health, United States of America

## Abstract

**Objective:**

We tested the hypothesis that patients with difficult asthma have an increased frequency of certain genotypes that predispose them to asthma exacerbations and poor asthma control.

**Methods:**

A total of 180 Caucasian children with confirmed asthma diagnosis were selected from two phenotypic groups; difficult (n = 112) versus mild/moderate asthma (n = 68) groups. All patients were screened for 19 polymorphisms in 9 candidate genes to evaluate their association with difficult asthma.

**Key Results:**

The results indicated that LTA4H A-9188>G, TNFα G-308>A and IL-4Rα A1727>G polymorphisms were significantly associated with the development of difficult asthma in paediatric patients (p<0.001, p = 0.019 and p = 0.037, respectively). Haplotype analysis also revealed two haplotypes (ATA haplotype of IL-4Rα A1199>C, IL-4Rα T1570>C and IL-4Rα A1727>G and CA haplotype of TNFα C-863>A and TNFα G-308>A polymorphisms) which were significantly associated with difficult asthma in children (p = 0.04 and p = 0.018, respectively).

**Conclusions and Clinical Relevance:**

The study revealed multiple SNPs and haplotypes in LTA4H, TNFα and IL4-Rα genes which constitute risk factors for the development of difficult asthma in children. Of particular interest is the LTA4H A-9188>G polymorphism which has been reported, for the first time, to have strong association with severe asthma in children. Our results suggest that screening for patients with this genetic marker could help characterise the heterogeneity of responses to leukotriene-modifying medications and, hence, facilitate targeting these therapies to the subset of patients who are most likely to gain benefit.

## Introduction

Asthma is one of the most common diseases worldwide [1]. It affects an estimated 300 million individuals, resulting in substantial morbidity, mortality, and health care utilisation. It has been estimated that about 4% of the British and North American population have asthma. Mortality from asthma occurs in approximately 0.4 per 100,000 and is currently estimated at about 1,500 per annum in the UK [2]. Asthma is not a curable disease, therefore, the goal of treatment is to control asthma symptoms and obtain a better quality of life for patients through maintaining normal or near normal lung function and preventing pulmonary exacerbations [3].

National and international treatment guidelines such as the British Thoracic Society and the Scottish Intercollegiate Guidelines Network [3], advocate the importance of anti-inflammatory agents in the management of chronic asthma symptoms. However, there is a subgroup of patients who are still insufficiently controlled on the maximal dose of asthma therapy, particularly in relation to inhaled corticosteroid (ICS) [4]. This subgroup of patients is known as difficult-to-treat asthma patients or in short patients with ‘difficult asthma’. There is no universally agreed definition for difficult asthma, although the term has been adopted by the European Respiratory Society (ERS) in 1999 [5]. According to the ERS, difficult-to-treat asthma includes “patients on high doses of control medication who either have or have not achieved control at this level of treatment” [6]. These patients are considered a source of significant concern as they suffer from continued impaired lung function and frequent exacerbations which predispose them to life threatening attacks and asthma-related death. In addition, they are at a higher risk of developing adverse drug effects, in particular due to high doses of steroid therapy (both inhaled and oral) [7].

Only one half of children referred to secondary or tertiary care clinics with apparent difficult asthma have true severe therapy resistant asthma (STRA). The remainder have difficult to treat asthma (DTA) but have not been instituted correct basic asthma management. This includes poor inhaler technique, inadequate medication adherence and exposure to on-going triggers of airways inflammation [4]. In addition to the multiple and complex factors that may contribute to uncontrolled symptoms, genetic predisposition is considered an important factor [8]. For example, available data from previous pharmacogenetic studies, suggests that as much as 70% of the variability in therapeutic responses to pharmacotherapy is genetically determined [9]–[11].

A number of polymorphisms in genes encoding for different enzymes, receptors and cytokines that are involved in asthma drug therapy and inflammation pathways, have been associated with asthma and/or asthma-related phenotypes in various studies [12]–[19]. These include genes encoding the β2-adrenergic receptor (ADRβ2), corticotrophin releasing hormone receptor 1 (CRHR1), leukotriene C4 synthase (LTC4S), leukotriene A4 hydrolase (LTΑ4H), interleukin-4 (IL-4), interleukin-13 (IL-13), interleukin-4 receptor α (IL-4Rα), tumour necrosis factor α (TNFα) and lymphotoxin α (LTA, alternative name: TNFβ). A key question, therefore, is whether subjects with certain genotypes fail to achieve asthma control and hence become classified as having difficult asthma.

The aim of the present study was to investigate whether patients with difficult asthma have an increased frequency of certain genotypes that predispose them to asthma exacerbations and poor asthma control. The presence of such an association will support the hypothesis that inappropriate treatment regimens, can lead to the development of a complex group of difficult asthmatics. As it is unlikely that only one genetic marker could significantly predict disease pathogenesis or drug response, 19 representative SNPs of relevant polymorphisms in nine candidate genes (ADRβ2, CRHR1, LTC4S, LTΑ4H, IL-4, IL-13, IL-4Rα, TNFα and LTA) and their common haplotypes were investigated in the present study in one ethnic group, a Caucasian population. This is the first study to examine the relationship between difficult asthma and these polymorphisms of interest in children.

## Methods

### Ethics statement

The study was approved by the Office for Research Ethics Committees in Northern Ireland (Ref no. 08/NIR02/18). Patients were included in the study only after their parents or legal guardians had been fully informed and had signed the study consent form. In addition, verbal assent was obtained from older children (≥6 years) before enrolment into the study.

### Study subjects

A total of 180 Caucasian children with confirmed asthma diagnosis were recruited into the study from 5 paediatric outpatient clinics in four different centres in Northern Ireland (the Royal Belfast Hospital for Sick Children, Cupar Street Asthma Clinic N&W Belfast, Craigavon Area Hospital and Ulster Hospital) and 3 primary care centres. Asthmatic children (under 16 years old) attending the clinics were classified into two: 1] Difficult to treat asthma, DTA (n = 112) and 2] mild/moderate asthma (n = 68) groups. DTA groups were recruited mainly from the hospital outpatient clinics while the majority of mild/moderate (M/M) asthma patients were recruited from the participating primary care centres. The classification of DTA was based on on-going asthma symptoms despite treatment with at least 800 micrograms (400 micrograms for children <5 years) of inhaled corticosteroid, ICS. Children over 5 years of age were taking additional long-acting beta 2 agonist while for those under 5 years a leukotriene antagonist was added mirroring steps 4 and 5 or the British Thoracic Society Guidelines (step 3/4 in children <5 years old) [3]. It is routine practice at the asthma clinics for inhaler technique to be checked, adherence to be assessed by child/parental questioning and for home trigger factors to be determined and avoidance advice given, but no home visits are arranged. The Mild/Moderate (MM) asthma classification was used for children whose asthma was readily controlled on a smaller dose of ICS.

### Study design

Buccal swabs were obtained from patients (one swab per individual) during the clinic visit. In the present study, SK-2 Isohelix buccal swabs (Ishohelix, Cell Projects Ltd, Kent, UK) were used for collecting cheek cells for the purpose of DNA extraction. Participants were advised not to consume any food or drink 30 minutes prior to sample collection. The samples were kept at −20°C for no longer than four weeks before DNA extraction.

Patient demographic details as well as clinical and medical characteristics were obtained directly from patients and/or their parents (through a structured questionnaire) and from their medical charts. Data collected for each patient included: age, gender, weight, age at diagnosis, prescribed medications and their dosages, passive smoking status, personal and family history of hay-fever and/or eczema, non-asthma related co-morbidity, records of medication side effects experienced and records of asthma exacerbations (defined as unscheduled clinic visits or hospitalisations within the previous 12 months).

### Genotyping the polymorphisms of interest

All patients were screened for 19 polymorphisms in nine candidate genes (ADRβ2, CRHR1, LTC4S, LTΑ4H, IL-4, IL-13, IL-4Rα, TNFα and LTA) to evaluate their association with difficult asthma. The polymorphisms selected in the present study were either functional SNPs or SNPs which have been reported and implicated with clinical relevance in the literature. For instance, A46>G and C79>G polymorphisms in the gene encoding the human β_2_-adrenoceptor have been shown to alter its function in the cardiovascular and respiratory systems [12], [20]–[21]. LTC4S A-444>C and LTA4H A-9188>G polymorphisms have been associated with altered response to leukotriene antagonists [13]–[14]. Furthermore, G35892>T, A38952>G and G54898>A polymorphisms in the CRHR1 gene were associated with alterations in the therapeutic responses to inhaled corticosteroids in asthmatic patients [15].

Other polymorphisms in genes related to T helper cell (Th) pathway have been associated with asthma and related phenotypes (including TNFα T-1031>C, C-863>A and G-308>A polymorphisms, LTA C804>A, and C-1111>T and G431>A polymorphisms in IL-13 gene) [17]–[19], [22]–[23]. In addition, polymorphisms in both IL-4 and IL4-Rα (C-590T and C-33>T in IL-4 and A223>G, A1199>C, T1570>C and A1727>G SNPs in IL4-Rα) have been associated with severity of asthma as measured by FEV_1_ and asthma exacerbations in recent studies [16]–[17], [24].

In the present study, genomic DNA was extracted from SK-2 swabs using the QIAmp® DNA mini kit protocol for buccal swabs (Qiagen, Hilden, Germany). The concentration of the extracted DNA was quantified using a NanoDrop 1000® spectrophotometer (Thermo Fisher Scientific, Delaware, USA) according to the manufacturer's instructions. Detection of the various SNPs of interest was carried out using the Sequenom® MassARRAY genotyping platform (iPlex assay, Sequenom®, Hamburg, Germany) and the validated TaqMan® genotyping assays obtained from Applied Biosystems (ABI, Foster City, USA).

The possibility of detecting the various SNPs of interest using the iPlex systems of the Sequenom® platform was assessed using the MassARRAY Assay Design software (Sequenom®, Hamburg, Germany). The highest plex level that could be achieved, however, was a bundle of 9 SNPs which included ADRβ2 A46>G, LTC4S A-444>C, LTA4H A-9188>G, CRHR1 A38952>G, CRHR1 G54898>A, IL-4 C-33>T, IL-4Rα T1570>C, IL-4Rα A1727>G and IL-13 C-1111>T SNPs. Approximately 15 ng of genomic DNA was used to genotype each sample. The DNA samples were amplified by multiplex polymerase-chain reactions (PCR), and the PCR products were then used for locus-specific single-base extension reactions. The resulting products were desalted and spotted into a 384-element SpectroCHIP array and then subjected to the MALDI-TOF mass spectrometry. SpectroTYPER software was used for the identification of SNP-specific signals and for automatic genotype calling.

The remaining 10 SNPs (ADRβ2 C79>G, CRHR1 G35892>T, IL-4 C-590>T, IL-13 G431>A, IL-4Rα A223>G, IL-4Rα A1199>C, TNFα T-1031>C, TNFα C-863>A, TNFα G-308>A and LTA C804>A) were genotyped using TaqMan® genotyping assays. The TaqMan® primer/probe set designed for each SNP allele was included in the kits purchased from Applied Biosystems (ABI, Foster City, USA). TaqMan® PCR reactions were performed in a 384-well format in a 5 µl reaction mixture containing 2.5 µl TaqMan® universal PCR master mix and using an amplification protocol of 95°C for 10 min, followed by 45 cycles of 92°C for 15 sec, then 60°C for 1 min. An allelic discrimination analysis was carried out using the ABI7900HT sequence detection system (ABI, Foster City, USA).

All sample plates contained cases, controls, blanks and duplicate samples. Quality control measures included independent double genotyping and, where available, comparison of our genotypes to those in the Hapmap database (http://www.hapmap.org/). All genotyping analysis was performed within the Genomic Core Facility, Queen's University Belfast.

### Statistical analysis

Statistical analysis was performed using SPSS® computer software (version 19; SPSS Inc, Chicago, IL, USA). Results were expressed as the mean and 95% confidence intervals or as frequencies (in the case of allele or haplotype carriers in patient groups). The difference in the clinical and medical characteristics between the two asthmatic groups (difficult and M/M) was investigated using the Chi-square (χ^2^) test (categorical variables) and Mann-Whitney *U*-test (non-parametric test for continuous variables).

All markers were tested for deviation from the Hardy-Weinberg Equilibrium (HWE) in both M/M and difficult asthmatic groups using a Chi-square (χ^2^) goodness-of-fit test, before analysis to evaluate the accuracy of genotyping. The difference in allele frequencies and genotype distribution of each polymorphism between the two asthmatic groups (difficult versus M/M) were compared using the Chi-square (χ^2^) test or the Fisher-Exact test as appropriate. Odds ratio (OR) were also calculated using binary logistic regression with 95% confidence intervals (CI). We considered two genetic models for genotype analysis: dominant (homozygote allele 1 vs. heterozygote plus homozygote allele 2) and recessive (homozygote allele 1 plus heterozygote vs. homozygote allele 2). Statistical significance was set at a p-value <0.05. Power analysis calculations to estimate the number of subjects needed to detect significant ORs assuming average allele frequencies in the control population was carried out using the statistical software package StatCalc (version 7.1.0.6; Epi Info™, Atlanta GA, USA).

Haplotype analysis was performed using the Haploview® programme (version 4.2) and blocks were identified using the confidence intervals method by Gabriel *et al.* [25]. Correction for multiple testing was performed using permutation correction (n = 100,000) by the Haploview program [26]. This approach corrects for multiple testing but takes into account the correlation between markers [26]. Finally, the presence of gene-gene interactions was determined using the likelihood algorithms implemented in the program UNPHASED [27]. Specifically, individual SNPs that were significantly associated with asthma severity after correction for multiple testing were chosen for further analysis of gene-gene interactions.

## Results

### Subject characteristics

A total of 180 children with asthma were enrolled in this study. These included 112 with difficult asthma and 68 with M/M asthma. More than half of the participants were male (64.4%) and the mean age was 9.1 years old. The majority of children with difficult asthma (90.2%) had at least one indicator of asthma exacerbation in the preceding 12 months; 76% had unscheduled GP or hospital visits, 34% had A&E attendances, 25% had hospital admissions and 76% had a rescue course of steroid therapy. On the other hand, only 30.9% of M/M asthma had at least one indicator of asthma exacerbation with no patients admitted to hospital in the preceding 12 months. Detailed demographic and clinical characteristics of subjects included in the study are presented in **Table 1**.

**Table 1:** Demographic and clinical characteristics of paediatric asthma patients (n = 180).

Characteristic	M/M asthma (n = 68)^a^	Difficult asthma (n = 112)^a^	P value
**Age at evaluation (years)^b^**	8.79±3.15 [2]–[15]	9.35±3.80 [1]–[16]	**NS**
**Gender (female)**	29 (42.6%)	35 (31.3%)	**NS**
**Smoking**			**NS**
**Never**	66 (98.5%)	105 (98.1%)	
**Ex-smoker**	0	0	
**Current smoker**	1 (1.5%)	2 (1.9%)	
**Personal history of hayfever/eczema**	42 (63.6%)	82 (74.5%)	**NS**
**Family history of asthma**	45 (67.2%)	86 (78.2%)	**NS**
**Family history of hayfever/eczema**	46 (69.7%)	63 (64.9%)	**NS**
**At least one indicator of asthma exacerbation in the last 12 months**	21 (30.9%)	101 (90.2%)	**<0.001**
**Unscheduled visit (GP, hospital)**	20 (29.4%) [0–2]	83 (75.5%) [0–14]	**<0.001**
**Accident & emergency attendance**	7 (10.3%) [0–2]	37 (34.3%) [0–10]	**<0.001**
**Hospital admission**	0	27 (24.5%) [0–10]	**<0.001**
**Rescue steroid course**	10 (14.7%) [0–1]	81 (75.7%) [0–15]	**<0.001**
**BTS step of asthma treatment (%)**			**<0.001**
**Step 1**	4 (5.9%)	0	
**Step 2**	31 (45.6%)	0	
**Step 3**	33 (48.5%)	0	
**Step 4**	0	74 (66%)	
**Step 5**	0	38 (33.9%)	
**Number of asthma drug categories^b^**	2.2±0.6 [0–3]	4.1±0.9 [3]–[7]	**<0.001**
**Use of home nebuliser**			**<0.001**
**No**	52 (89.7%)	37 (33%)	
**Yes (as needed)**	6 (10.3%)	56 (50%)	
**Yes (regular)**	0	19 (17%)	
**Maintenance oral steroid**	0	14 (12.6%) [2.5–50]	**0.002**
**BDP equivalent of daily ICS (µg)^b^**	332.0±180.5 [100–800]	1247.6±897.5 [200–4000]	**<0.001**

BTS: British Thoracic Society; GP: general practitioner; BDP: beclomethasone diproprionate; ICS: inhaled corticosteroid.

a in some cases data were missing, i.e. percentage values correspond to numbers of patients for which full data were available.

b data presented as mean±SD; [range].

Since most polymorphisms of interest have a minor allele frequency (MAF) of 15–33% in the Caucasian population, power analysis showed that the number of recruited patient in this study provided a sufficient sample size to detect an OR of 2.57 (with 80% power at 5% α-level) for an increased frequency of risk alleles in patients with difficult asthma as compared to patients with mild/moderate asthma assuming an average MAF of 23% in the population (19% increase will be detected if MAF = 15% and 21% increase if MAF = 33%).

### Genotype and allele frequencies

Allele and genotype frequencies for each SNP selected for investigation are shown in **Table 2**. All SNPs were in Hardy-Weinberg Equilibrium (HWE) (p>0.01) in both M/M and difficult asthmatic subgroups apart from ADRβ2 C79>G (p = 0.004 in difficult asthmatics). The latter SNP was, therefore, excluded from analysis in the present study. All of the remaining SNPs had a call rate >95% and a genotyping accuracy >99%.

**Table 2:** Allele and genotype frequencies of the genetic variants of interest in the study cohort (n = 360 alleles).

Genetic variant	dbSNP#	SNP type‡	Variant allele frequency (%)	Heterozygotes (%)
***ADRβ2 A46*** **>** ***G***	rs1042713	missense (Arg16>Gly)	36.4%	38.3%
***CRHR1 G35892*** **>** ***T*** †	rs242941	synonymous	32.5%	40.6%
***CRHR1 A38952*** **>** ***G*** †	rs242939	synonymous	8.1%	16.1%
***CRHR1 G54898*** **>** ***A*** †	rs1876828	synonymous	19.3%	30.7%
***LTC4S A-444*** **>** ***C***	rs730012	synonymous	31.8%	46.9%
***LTA4H A-9188*** **>** ***G*** †	rs2660845	synonymous	27.5%	43.8%
***IL-4 C-590*** **>** ***T***	rs2243250	synonymous	11.0%	19.8%
***IL-4 C-33*** **>** ***T***	rs2070874	synonymous	11.5%	20.7%
***L-13 C-1111*** **>** ***T***	rs1800925	synonymous	17.3%	26.8%
***IL-13 G431*** **>** ***A***	rs20541	missense (Arg130>Glu)	15.7%	26.5%
***IL-4Rα A223*** **>** ***G***	rs1805010	missense (Ile50>Val)	42.8%	47.8%
***IL-4Rα A1199*** **>** ***C***	rs1805011	missense (Glu375>Ala)	9.2%	17.3%
***IL-4Rα T1570*** **>** ***C***	rs1805015	missense (Ser478>Pro)	15.6%	28.9%
***IL-4Rα A1727*** **>** ***G***	rs1801275	missense (Gln551>Arg)	18.1%	32.8%
***LTA C804*** **>** ***A***	rs1041981	missense (Thr60Asn)	39.9%	47.5%
***TNFα T-1031*** **>** ***C***	rs1799964	synonymous	21.9%	31.5%
***TNFα C-863*** **>** ***A***	rs1800630	synonymous	17.6%	27.4%
***TNFα G-308*** **>** ***A***	rs1800629	synonymous	24.0%	32.7%

† Numbering was based on RefSeqGene mapping, NCBI database.

‡ The polymorphism is termed missense if the nucleotide change alters the amino acid sequence, termed synonymous otherwise.

### Association between genetic variants and difficult asthma

Possible associations between difficult asthma and the presence of risk alleles for any of the studied polymorphisms were examined (**Table 3**). The results indicated that allele A of LTA4H A-9188>G (rs2660845), allele G of TNFα G-308>A (rs1800629) and allele A of IL-4Rα A1727>G (rs1801275) were all significantly associated with difficult asthma in paediatric patients after adjusting for multiple testing (p<0.001, p = 0.019 and p = 0.037, respectively).

**Table 3:** Association analysis between the various polymorphisms of interest and asthma phenotypes (difficult versus M/M asthma).

Genetic variant	Associated allele	Difficult Asthmatics	M/M Asthmatics	p-value	p-value (adjusted)†
***ADRβ2 A46*** **>** ***G***	A	37.5%	34.6%	0.57	-
***CRHR1 G35892*** **>** ***T***	T	34.4%	29.4%	0.32	-
***CRHR1 A38952*** **>** ***G***	A	93.8%	89.0%	0.11	-
***CRHR1 G54898*** **>** ***A***	G	82.0%	78.7%	0.44	-
***LTC4S A-444*** **>** ***C***	C	33.3%	29.4%	0.43	-
***LTA4H A-9188*** **>** ***G***	A	79.5%	61.0%	1×10^−4^	0.0007
***IL-4 C-590*** **>** ***T***	C	89.1%	88.8%	0.93	-
***IL-4 C-33*** **>** ***T***	C	88.7%	88.2%	0.88	-
***IL-13 C-1111*** **>** ***T***	T	18.0%	16.2%	0.65	-
***IL-13 G431*** **>** ***A***	A	15.9%	15.2%	0.86	-
***IL-4Rα A223*** **>** ***G***	A	57.6%	56.6%	0.85	-
***IL-4Rα A1199*** **>** ***C***	A	93.3%	86.6%	0.033	0.14
***IL-4Rα T1570*** **>** ***C***	T	87.9%	78.7%	0.018	0.091
***IL-4Rα A1727*** **>** ***G***	A	86.2%	75.0%	0.007	0.037
***LTA C804*** **>** ***A***	C	62.5%	56.0%	0.22	-
***TNFα T-1031*** **>** ***C***	C	24.6%	17.4%	0.11	-
***TNFα C-863*** **>** ***A***	A	20.7%	12.5%	0.047	0.22
***TNFα G-308A***	G	80.8%	66.9%	0.004	0.019

† Adjusted p-values were considered when the initial uncorrected values were <0.05.

A further evaluation of the genotype distribution of these polymorphisms showed that carriers of the AA genotype of LTA4H A-9188>G or IL-4Rα A1727>G polymorphisms and carriers of the GG genotype of TNFα G-308>A polymorphism were at an increased risk of having difficult asthma when compared with carriers of other genotypes of these polymorphisms (OR = 3.0; 95%CI = 1.6–5.7; p = 0.001, OR = 2.4; 95%CI = 1.3–4.6; p = 0.006 and OR = 2.2; 95%CI = 1.1–4.1; p = 0.019, respectively).

Analysis of the allelic distribution among patients according to their asthma severity also indicated nominally significant associations of allele A of IL-4Rα A1199>C, allele T of IL-4Rα T1570>C and allele A of TNFα TNFα C-863>A with asthma severity (p = 0.033, p = 0.018 and p = 0.047, respectively). However, these associations were not significant after correction for multiple testing.

### Linkage disequilibrium and haplotype association analysis

**Figure 1:**
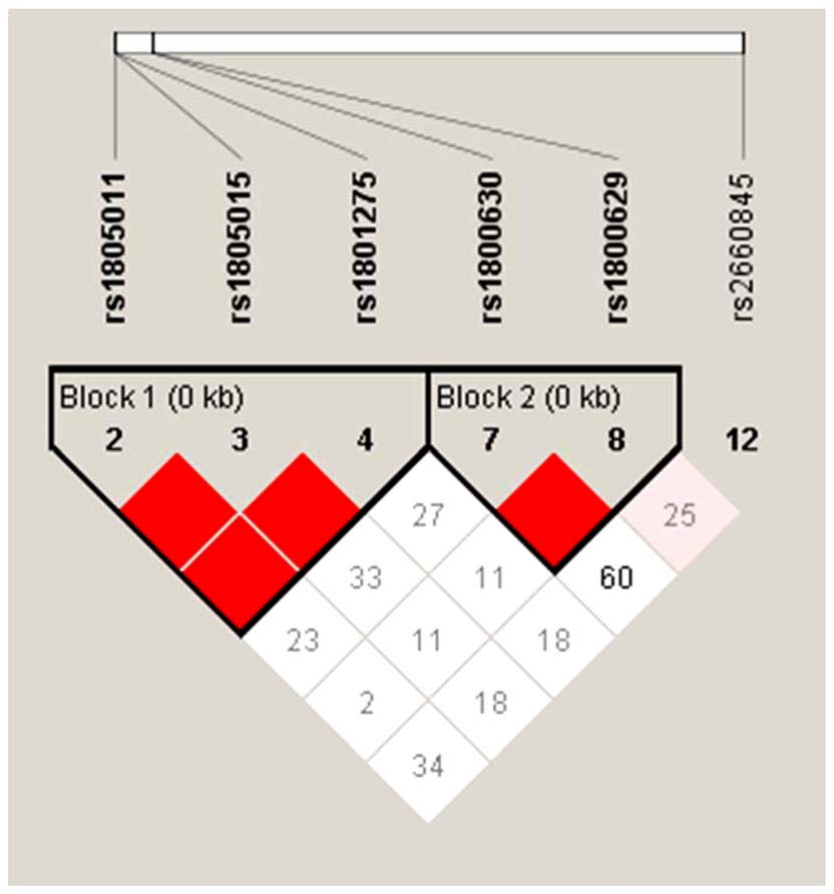
Linkage disequilibrium (LD) structure of the examined genetic loci of interest in the study population. The haplotype blocks were generated by Haploview using the default algorithm [21]. Values in the boxes are D' measures, indicating extent of LD between two SNPs. Boxes without numbers have D' = 1.

In order to investigate the combined effect of more than one SNP, haplotype analysis was carried out for the SNPs that showed significant or nominally significant association with difficult asthma. The results of haplotype analysis are presented in **Table 4**. There was strong linkage disequilibrium (D' = 1) between the three selected IL-4Rα polymorphisms (A1199>C, T1570>C and A1727>G) and between the two TNFα polymorphisms (C-863>A and G-308>A). These five SNPs formed two blocks, one in each gene. LTA4H A-9188>G, however, did not segregate with any of these SNPs, **Figure 1**. Two haplotypes (ATA in the first block, chromosome 16 and CA in the second block, chromosome 6) showed significant association with difficult asthma, including after correction for multiple testing (p = 0.04 and p = 0.018, respectively). The ATA haplotype was the most common haplotype in block 1 (81.9%), whereas CA was the next most frequent in block 2 (24.2%) following GC as the most frequent (58.1%).

**Table 4:** Association of the examined haplotypes with the risk of developing difficult asthma in children.

Haplotype	Frequency, % (Study Cohort)	Frequency ratio, % (Difficult:M/M)	p-value	p-value (adjusted)†
**Block 1:** ***IL-4Rα*** **(** ***A1199*** **>** ***C*** **,** ***T1570*** **>** ***C*** and ***A1727*** **>** ***G*** **)**				
ATA	81.9%	86.2%∶75.0%	0.007	0.040
CCG	9.2%	6.7%∶13.2%	0.037	0.187
ACG	0.6%	5.4%∶8.1%	0.304	-
ATG	2.5%	1.8%∶3.7%	0.265	-
**Block 2:** ***TNFα*** **(** ***C-863*** **>** ***A*** **and** ***-308A*** **)**				
CG	58.1%	60.1%∶54.9%	0.338	-
CA	24.2%	19.2%∶32.6%	0.004	0.018
AG	17.6%	20.7%∶12.5%	0.046	0.238

† Adjusted p-values were considered when the initial uncorrected values were <0.05.

Furthermore, another two haplotypes, CCG (the second most frequent haplotype in block 1) and AG (the least frequent haplotype in block 2) were more prevalent in children with M/M asthma compared to those with difficult asthma (p = 0.037 and p = 0.046, respectively). However, these associations did not reach statistical significance after correction for multiple testing.

### SNP-SNP interactions

To test for potential interaction between the polymorphisms on affection status, polymorphisms showing the strongest association with difficult asthma were selected, i.e. LTA4H A-9188>G, TNFα G-308>A and IL-4Rα A1727>G. No evidence of gene-gene interaction was observed between pairs of associated markers (p>0.05 for all two-way interactions using UNPHASED likelihood algorithm). Furthermore, the three SNPs, LTA4H A-9188>G, TNFα G-308>A and IL-4Rα A1727>G, remained significant after conditioning on the effect of each of the other associated SNPs. This suggests that each of the 3 SNPs is exerting an independent effect.

## Discussion

Asthma medications comprise a substantial portion of health-care expenditure. Despite new therapies becoming available in all developed countries, there is still a significant number of non-responders [9], [28]. Evidence suggests that genetic factors may mediate a large part of such heterogeneity in response to each of the three major classes of asthma medications [29].

β2-agonists are the most commonly used class of medication in the treatment of asthma worldwide [30]. β2-agonists produce bronchodilatation (increase FEV1) in asthmatics by binding to and directly stimulating β2-adrenoceptors on airway smooth muscle cells. A number of polymorphisms in the gene encoding the human β2-adrenoceptor (e.g. A46>G and C79>G polymorphisms which modify the amino acid sequence of the receptor into Arg16>Gly and Gln27>Glu, respectively) have been known to alter its function in the cardiovascular and respiratory systems. In the airway, the presence of the homozygous Arg16 genotype (wild) has been shown to confer relative protection against β2-adrenoceptor down-regulation by endogenous catecholamines. This explains the greater bronchodilator response to acute β2-agonist therapy in Arg16 homozygotes but results in a diminished therapeutic response to prolonged or chronic β2-agonist therapy and reverses the benefits from the regular use of both short and long acting β2-agonists in these patients [12], [20]–[21]. Conversely, down-regulation of β2-adrenoceptors in patients with the Glu27 genotype (variant) has been shown to be attenuated in comparison with those with the Gln27 genotype (wild). Studies have shown an increased likelihood of exacerbations among children and adults with mild-moderate asthma who were homozygous for the Arg16 genotype when regularly dosed with short or long acting β2-agonists [21], [31]–[34]. The development of difficult asthma and its association with the Arg16/Arg16 genotype or other genotypes, however, has not been investigated before. In the present study, we demonstrate no association between these SNPs and difficult asthma.

The second class of asthma drugs for which pharmacogenetics could contribute to variability in treatment response is leukotriene antagonists. Leukotrienes are a family of potent bronchoconstrictors which are produced in the airways of asthmatics. Of the many enzymes involved in the formation of leukotrienes, polymorphisms in two (LTC4S, and LTA4H) have been associated with altered response to leukotriene antagonists [13]. Of potential interest, LTA4H A-9188>G SNP has been associated with a fourfold increase in the risk of asthma exacerbations while LTC4S A-444>C SNP has been associated with 76% reduction in exacerbations as shown in a recent study [35]. Observational data in difficult asthma cohorts, suggests that this class of agent has less efficacy in this population, and again raises the possibility that a non-responsive phenotype is found in subjects with difficult asthma.

Interestingly, the present study revealed strong evidence for the involvement of LTA4H A-9188>G polymorphism in the development of difficult asthma in children. Current findings suggest that carriers of the AA genotype are at 3-times higher risk of developing difficult asthma than carriers of the GG genotype. Relative to other genes, LTA4H has not been thoroughly examined in genetic association studies and the number of investigations analysing the association of polymorphisms in this gene with asthma-related phenotypes is limited [13]–[14], [35]. To our knowledge, this is the first study to investigate the association between this gene and severe asthma in children.

The third class of medication used in asthma is the glucocorticoids. Glucocorticoids are the most potent anti-inflammatory agents in current routine use in the treatment of asthma. In general, glucocorticoids activate anti-inflammatory genes and repress the expression of pro-inflammatory cytokines [36]. Hence, they attenuate airway inflammation and hyper-responsiveness in asthmatic patients. Glucocorticoids are very effective in those who respond. However, the hallmark of difficult asthma is relative steroid resistance, resulting in the requirement for higher doses, with resultant undesirable side-effects [37]. Many mechanisms have been postulated to explain this relative steroid resistance, including defects in the CRHR1 receptor, because it is a major regulator of glucocorticoid synthesis. A recent study showed that genotype differences in the CRHR1 gene were associated with alterations in the therapeutic responses to inhaled corticosteroids in asthmatic patients [15]. In the present study, however, there was no association identified between the same three polymorphisms discussed earlier (G35892>T, A38952>G and G54898>A) and difficult asthma.

Numerous other genes have been associated with asthma and related phenotypes, including those related to T helper cell (Th) pathways (IL-4, IL-13, IL-4Rα, TNFα and LTΑ) [16]-[17], [38].

The TNF superfamily which belongs to the Th1 pathway [39], has been shown to play an important role in the immune response and triggering of inflammation which is of paramount importance in the pathophysiology of asthma. TNFα and LTA genes are clustered together on chromosome 6p21.3. Variations in these genes have been associated with asthma through both case-control and family-based studies, performed in diverse populations [18]–[19], [40]–[42]. Of particular interest is the TNFα G-308>A SNP which has been associated with increased bronchial hyper-reactivity [43] and wheezing [44]–[45]. In the present study, the G allele of TNFα G-308>A SNP conferred a significant risk of developing difficult asthma in children. Furthermore, the G-308>A polymorphism was in strong linkage disequilibrium with C-863>A and carriage of the CA haplotype (TNFα -863C and -308A) has been significantly associated with difficult asthma, even after correction for multiple testing.

In addition, polymorphisms in IL4-Rα (related to Th2 pathway) have been associated with severity of asthma in the present study; nominally significant associations were found between A1199>C, T1570>C polymorphisms and difficult asthma and A1727>G was strongly associated with difficult asthma in children after adjustment for multiple testing. This is supported by previous studies which observed significant associations between IL4-Rα polymorphisms (A1199>C, T1570>C and A1727>G) and asthma-related phenotypes [46]–[48]. In these studies, patients who carried the A allele of the rs1801275 polymorphisms showed elevated total serum IgE levels [46]–[47] and an increased risk of allergic asthma [48]. The latter association was particularly observed in patients with persistent asthma. In the present study, the haplotype ATA (of IL4-Rα A1199>C, T1570>C and A1727>G polymorphisms) was significantly more frequent in children within the difficult asthma group.

In addition, polymorphisms in IL-4 (related to Th2 pathway) have been associated with severity of asthma as measured by FEV1 [16]. Of interest, is the C-590>T SNP which has been associated, in a recent study, with fatal or near-fatal asthma in adults [24]. Previous studies have also shown associations of C-1111>T and G431>A polymorphisms in the IL-13 gene with various features of the asthmatic phenotype [22]–[23]. In the present study, however, none of these SNPs were significantly associated with difficult asthma in our paediatric population.

One potential limitation of our study is that in the difficult asthma group we were unable to confidently separate out children with true severe therapy resistant asthma from those with difficult to treat asthma for other causes. This would have required that all such children would have been screened through an invasive difficult asthma protocol and been observed for 3–6 months to have adhered to optimised therapy [4]. Future studies would benefit from this stratification. However, our original hypothesis was best served by studying a wider group of children with difficult asthma as this would include children who may have stopped taking medication as it truly was not working. Another limitation of the study is the relatively small cohort size (particularly the M/M asthma group) which likely contributed to the wide CI ranges and insufficient power (<80%) to detect ORs of less than 2 for association of difficult asthma and potential SNPs particularly those with MAF less than 15%. The present findings should, therefore, be interpreted with caution as it is not possible to draw definitive conclusions on the association between SNPs with MAF<23% and asthma phenotypes without validation in a separate cohort. Validation of the findings in a larger population is warranted to examine the potential importance of these polymorphisms.

In conclusion, we have identified multiple SNPs and haplotypes in LTA4H, TNFα and IL4-Rα genes that constitute risk factors for the development of difficult asthma in children and demonstrate combined effects which confer greater risk than that obtained for any SNP individually. Functional studies focusing on the potential clinical implications of these findings are warranted. For example, our results suggest that screening for the associated genetic markers may identify individuals at risk of developing difficult asthma. Furthermore, our data suggest that LTA4H genetic variations could play an important role in the wide inter-patient variability of responses to leukotriene antagonists. A greater understanding of the molecular mechanisms underlying the effect of these genetic variations could provide insight into the overall mechanisms that cause susceptibility to difficult asthma and, hence, lead to new therapeutic opportunities for the treatment of this complex disease in children. It could also help characterise the heterogeneity of responses to leukotriene-modifying medications and facilitate targeting such therapies to the subset of asthmatic patients who are most likely to gain benefit.
